# The cumulative incidence of atrial fibrillation in the hospitalized medical patient: a systematic review and meta-analysis

**DOI:** 10.1093/ehjopen/oeag080

**Published:** 2026-05-22

**Authors:** Hani Essa, Ashwin Balu, Gregory Y H Lip, Ingeborg Welters

**Affiliations:** Department of Cardiovascular and Metabolic Medicine, Institute of Life Course and Medical Sciences, William Henry Duncan Building, University of Liverpool, Liverpool, L7 8TX, UK; University Hospitals of Liverpool Group, Royal Liverpool University Hospital, Mount Vernon Street, Liverpool, L7 8XP, UK; Liverpool Centre for Cardiovascular Science at University of Liverpool, Liverpool John Moores University and Liverpool Heart & Chest Hospital, 6 West Derby Street, Liverpool, L7 8TX, UK; Department of Cardiovascular and Metabolic Medicine, Institute of Life Course and Medical Sciences, William Henry Duncan Building, University of Liverpool, Liverpool, L7 8TX, UK; University Hospitals of Liverpool Group, Royal Liverpool University Hospital, Mount Vernon Street, Liverpool, L7 8XP, UK; Liverpool Centre for Cardiovascular Science at University of Liverpool, Liverpool John Moores University and Liverpool Heart & Chest Hospital, 6 West Derby Street, Liverpool, L7 8TX, UK; Liverpool Centre for Cardiovascular Science at University of Liverpool, Liverpool John Moores University and Liverpool Heart & Chest Hospital, 6 West Derby Street, Liverpool, L7 8TX, UK; Department of Clinical Medicine, Aalborg University, Selma Lagerløfs Vej 249, 9260 Gistrup, Aalborg, Denmark; Medical University of Bialystok, ul. Jana Kilińskiego 1, 15-089 Białystok, Poland; Department of Cardiovascular and Metabolic Medicine, Institute of Life Course and Medical Sciences, William Henry Duncan Building, University of Liverpool, Liverpool, L7 8TX, UK; University Hospitals of Liverpool Group, Royal Liverpool University Hospital, Mount Vernon Street, Liverpool, L7 8XP, UK; Liverpool Centre for Cardiovascular Science at University of Liverpool, Liverpool John Moores University and Liverpool Heart & Chest Hospital, 6 West Derby Street, Liverpool, L7 8TX, UK

**Keywords:** New-onset atrial fibrillation, Sepsis, Pneumonia, Critical illness, Pulmonary embolism

## Abstract

New-onset atrial fibrillation (NOAF) is the most common cardiac arrhythmia occurring in hospitalized patients. NOAF confers a significant risk of cardiovascular morbidity and mortality in the form of symptomatic tachyarrhythmia, heart failure, stroke and sudden cardiac death. It is unclear what the cumulative incidence of NOAF is in the hospitalized medical (non-surgical) patient. MEDLINE and Embase were searched for studies published between 01 January 2000 and 10 March 2026. All studies reporting the cumulative incidence of NOAF in hospitalized medical (non-surgical) patients were included. The pooled cumulative incidence of NOAF, 95% confidence intervals (CI), and 95% prediction intervals (PI) were computed using a random-effects model. The inconsistency index (*I*^2^) was calculated to measure heterogeneity. Subgroup analyses were also performed. A study protocol was registered with the PROSPERO database of systematic reviews (CRD42024626333) A total of 10 323 articles were identified from our searches, and of these 62 met the inclusion criteria. The cumulative incidence of NOAF across all studies was 99 474 (crude incidence 2.4%) out of 3 608 663 patients. The pooled cumulative incidence of NOAF was 9% (95% CI: 7–11%), with substantial heterogeneity (*I*^2^ = 99.9%) and a wide prediction interval (1–42%). In meta-regression, ICU setting (OR 2.46, 95% CI: 1.22–5.00; *P* = 0.013) and prospective study design (OR 1.73, 95% CI: 1.01–2.95; *P* = 0.044) were independently associated with higher NOAF incidence, while year of publication and use of administrative data were not significant. Subgroup analyses demonstrated consistently higher incidence in prospective and critically ill populations. The cumulative incidence of NOAF in patients hospitalized for acute medical illness varies significantly dependent on disease severity and methods to detect it. Attempts to pool data across studies are limited by differences in study design, patient populations and disease severity.

## Graphical Abstract

**Graphical Abstract:**
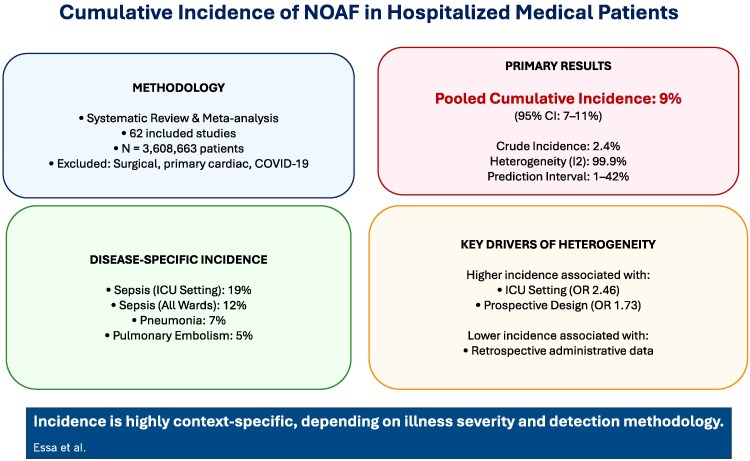

## Introduction

New-onset atrial fibrillation (NOAF) within the hospital setting is usually secondary to transient precipitants. It is widely recognized that acute stress, including but not limited to alcohol misuse (‘holiday heart syndrome’), acute myocardial ischaemia or infarction, pulmonary embolism, pneumonia or sepsis can induce NOAF.^1–6^ This form of atrial fibrillation (AF) is often a transient phenomenon that resolves with successful treatment of the stressor. Historically, this was viewed as distinct and ‘reversible’ entity with a greater emphasis placed on identifying and managing triggers (i.e sepsis) rather than empirical management of AF. Older consensus guidelines advocated that treatment of an underlying reversible precipitant may ‘terminate the arrhythmia without recurrence’.^7^ Therefore, it was standard practice in this patient cohort to not routinely offer treatment for AF.

Data accumulated from long-term follow-up of this cohort of patients demonstrated that they remained at significantly elevated risk of cardiovascular disease, death and stroke, comparable to otherwise age matched patients with permanent AF.^8^ Furthermore, a significant proportion of these patients experience AF recurrence, suggesting that NOAF is not a transient phenomenon.^6^ Increasingly, these episodes are now thought to represent the first episode of paroxysmal AF in this population. Hence, there is now a generalized agreement for anticoagulation and active management of AF in this cohort.^9^ However, certain exceptions do apply where consensus has been more difficult to achieve, chiefly in AF acquired during critical or severe acute illness.^10^ NOAF in the critically and acutely unwell population remains poorly understood. Commonly NOAF not related to established cardiac conditions is regarded as a precipitator of critical illness/deterioration in acute conditions. There is an emerging body of evidence to suggest that factors such as structural and valvular heart disease are not as strongly associated with the critically and acutely unwell population.^10^ Indeed, it is believed that rapid cardiac remodelling and fibrosis in combination with autonomic dysregulation plays a bigger role in this population.^1,11,12^ Hence there is considerable difference in practice between intensivists regarding anticoagulation, with most not routinely employing validated risk scores in their patients.^13,14^ Similarly, patients undergoing percutaneous coronary artery angiography for acute myocardial infarction often experience transient AF during coronary instrumentation.^15^ However, anticoagulation is commonly not offered and there remains no consensus on how best to manage AF in this context.^16^

It is unclear what the cumulative incidence of NOAF is in hospitalized medical patients. There are limited data on this topic with a previous systematic review in 2019 identified thirty-six studies reporting the cumulative incidence of NOAF. The data were very heterogeneous with reported cumulative incidence of 1–44%, and given the substantial heterogeneity, the authors chose to not pool results.^17^ Given the increase in studies reporting the cumulative incidence of NOAF over the past few years and the increasing use of telemetry in hospital it is justifiable to perform a systematic review to better answer this question.

## Methods

**Figure 1:**
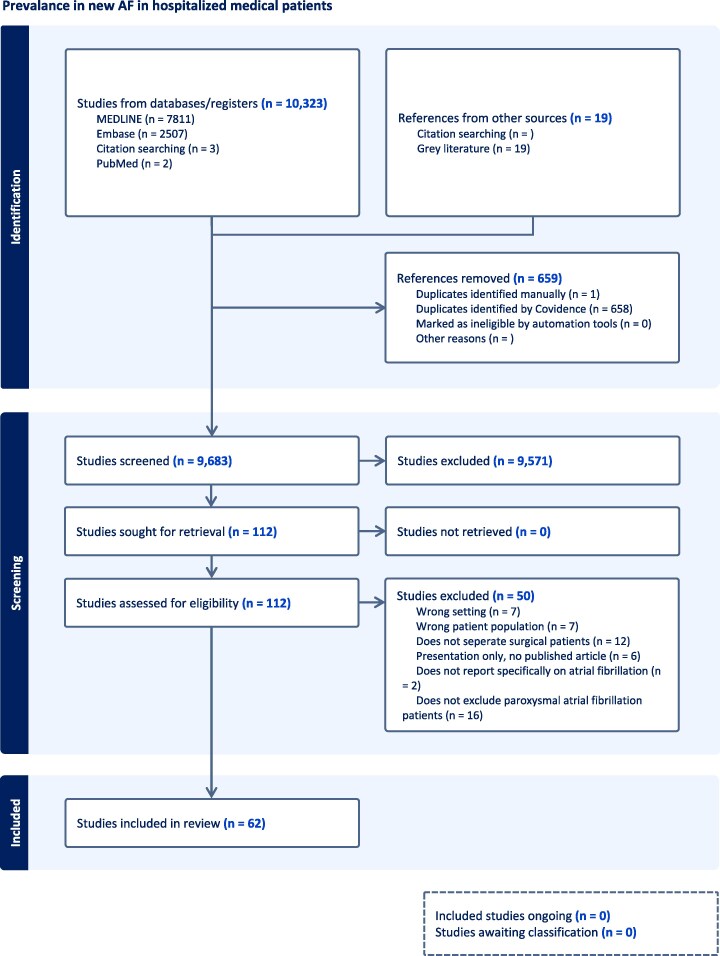
The systematic literature search across four databases (MEDLINE, Embase, PubMed, and Citation searching) and grey literature sources yielded 10 323 initial records. After the removal of duplicates (*n* = 659) and title/abstract screening, 112 reports were assessed for full-text eligibility. A total of 62 studies met the inclusion criteria and were included in the final meta-analysis. Reasons for exclusion at the full-text stage (*n* = 50) are specified within the diagram. The flow diagram was generated using Covidence systematic review software and adapted from the PRISMA 2020 flow diagram template, licensed under CC BY 4.0.

This review aims to systematically assess the cumulative incidence of NOAF in patients hospitalized for acute medical (non-surgical) illness. The review was conducted using established systematic review methodology and is reported in accordance with the Preferred Reporting Items for Systematic reviews and Meta-Analyses (PRISMA) guidelines (*Figure 1*) and was registered with the PROSPERO database of systematic reviews (CRD42024626333)

### Eligibility criteria

To be eligible for inclusion, articles had to report on adults hospitalized for a medical condition without a known history of AF. Studies were excluded if they focused on surgical conditions, stroke, primary cardiac condition (e.g. acute coronary syndrome, heart failure, or primary arrhythmia), or COVID-19, as COVID-19 represents a distinct and evolving disease entity, with the reported incidence of AF varying across different phases of the pandemic, likely reflecting changes in viral variants, vaccination status and treatment strategies. Studies reporting on a mixed population of medical and surgical patients were included only if it was possible to separately extract data for medical patients.

### Outcomes

The primary outcome assessed was cumulative incidence of NOAF. We did not prespecify any secondary outcomes.

### Search strategy

The following electronic databases were searched: Medical Literature Analysis and Retrieval System Online (MEDLINE), Excerpta Medica Database (EMBASE). Both databases were searched for articles published between 01 January 2000 and 24 November 2024 (search date) and were updated on 10 March 2026. Search terms and index terms AF and associated medical conditions including but not limited to sepsis, pneumonia, alcohol excess, falls, urinary tract infection and pulmonary embolism (Appendix 1). Only full-text articles were included. Searches were restricted to English-language publications from the year 2000 onwards to exclude older articles which may be less relevant and or employ outdated methodology or technology.

### Study selection and data extraction

Two researchers (HE, the author, and AB, fellow research student) independently assessed the suitability of papers for inclusion against the eligibility criteria. Any disagreements were resolved through consensus. Two researchers (HE and AB) independently extracted relevant data from the papers using a standardized tabulated data extraction form. Any disagreement was resolved through consensus.

### Risk of bias assessment

Whilst there is no accepted guideline for reporting risk of bias in cumulative incidence studies, we used a modified version (see Supplementary material online, *Table S1*) of the Newcastle–Ottawa Scale (NOS) for cohort studies.^18^

### Statistical analysis

We performed a random-effects meta-analysis using the Hartung-Knapp adjustment, which assigns relatively greater weight to smaller studies. This approach was used to improve the robustness of the results in the presence of heterogeneity and may shift pooled estimates towards the results of smaller studies compared with simple aggregate calculation. The 95% confidence interval (95% CI) was calculated utilizing the normal approximation method for proportions. The logit transformation was applied to stabilize variances, and results were back transformed to the proportion scale for interpretation. We calculated 95% prediction intervals (PI) which represent a range of values that predicts the effect size of a new potential study and provide a more comprehensive evaluation of heterogeneity.^19^ Statistical heterogeneity was assessed using the I^2^ statistic, with the following thresholds: <25% indicating low heterogeneity, 25–75% moderate, ≥75% considerable heterogeneity.^20^ To investigate for potential source of heterogeneity, we also performed subgroup analyses according to investigated condition (i.e. pneumonia, pulmonary embolism, sepsis, medical wards) and study setting [i.e intensive care unit (ICU)] and study type (i.e prospective Vs retrospective). Subgroup analyses were prespecified in the study protocol; however, the specific subgroup variables were not fully defined *a priori* and were refined *post hoc* based on data availability and clinical relevance across included studies. Differences between subgroups were assessed using a χ^2^ test for subgroup differences (Cochran’s Q), as implemented within the random-effects meta-analysis framework. A leave-one-out sensitivity analysis was performed to assess the influence of individual studies on the pooled estimate. Data synthesis and statistical analysis were supported using R version 4.4.1 (R foundation for statistical computing, Vienna, Austria) with the use of ‘meta’, ‘metafor’ and ‘dmetar’ packages.^21^

## Results

The searches identified 10 323 studies; additional 19 articles were identified through a grey literature (OpenGrey, Bielefeld Academic search engine) search. A total of 659 citations were removed as duplicates leaving 9683 articles for screening. Reasons for exclusion at the abstract and full-text stages are provided in *Figure 1*. Overall, a total of 62 articles were included in the review (*Table 1*).

**Table 1:** Characteristics of included studies

Author, Year	Setting	Condition	Nature of study	Total new AF	Control	Total patients	% new AF	AF age^a^	Control age^a^	Female (NOAF)	Female (control)	In-hospital mortality (AF)	In-hospital mortality (control)
Yang, 2026^22^	ICU	Sepsis	ROS	123	2000	2123	5.8%	72	62	48 (39%)	830 (41.5%)	25 (20.3%)	180 (9%)
Narváez, 2026^23^	ICU	Sepsis	ROS	171	1185	1356	12.6%	71	60	72 (42%)	534 (45%)	94 (55%)	435 (37%)
Huang, 2025^24^	ICU	Sepsis	ROS, ADB	4904	31 918	36 822	18.2%	N/A	N/A	N/A	N/A	N/A	N/A
Huo, 2025^25^	ICU	Sepsis	ROS, ADB	7691	24 506	32 197	23.9%	78.3	69.5	3640 (47.3%)	12 780 (52.1%)	2292 (29.8%)	2299 (9.4%)
Chai, 2025^26^	ICU	Sepsis	ROS	137	286	423	32.4%	67	64	53 (38.7%)	112 (39.1%)	N/A	N/A
Rottmann, 2024^27^	ICU	Medical patients	ROS	87	393	480	18.10%	74	73	31 (35.6%)	146 (37.2%)	24 (27.6%)	48 (12.2%)
Liu, 2024^28^	Medical ward	Sepsis	ROS, ADB	21 327	1 403 728	1 425 055	1.5%	N/A	N/A	N/A	N/A	4832 (22.6%)	172 580 (12.3%)
Myers, 2024^29^	Medical ward	Sepsis	ROS, ADB	3992	78 756	82 748	4.8%	N/A	N/A	N/A	N/A	N/A	N/A
Liang, 2024^30^	Medical ward	PE	ROS	29	703	732	4.00%	N/A	N/A	N/A	NA	N/A	N/A
Djuric, 2023^31^	Medical ward	PE	ROS, ADB	138	1342	1480	9.30%	72	61	78 (56.5%)	715 (48.3%)	48 (18.6%)	114 (8.5%)
Aiwa, 2022^32^	Medical ICU	Medical patients	ROS	29	242	271	10.70%	N/A	N/A	N/A	N/A	N/A	N/A
Li, 2022^33^	Medical ward	Sepsis	ROS	269	2223	2492	10.8	65.8	58.48	31 (35.6%)	146 (37.2%)	81 (30.1%)	457 (20.6%)
DeMiguel-Yanes, 2022^34^	Medical ward	Pneumonia	ROS, ADB	2136	28 936	31 072	5.50%	72.5	70	679 (31.8%)	12 753 (44.1%)	765 (35.8%)	10 382 (35.8%)
Søgaard, 2022^6^	Medical ward	Pneumonia	ROS, ADB	11 107	281 944	293 051	3.80%	N/A	N/A	N/A	N/A	N/A	N/A
Mcintyre, 2021^35^	Mixed ICU	Medical patients	PS	34	124	158	21.50%	N/A	N/A	N/A	N/A	N/A	N/A
Brunetti, 2021^36^	Medical ICU	Medical patients	ROS, ADB	241	1993	2234	10.70%	67.5	57	108 (44.8%)	914 (45.9%)	51 (21.2%)	206 (10.3%)
Liu, 2021^37^	Medical ward	PE	ROS	54	536	590	9.10%	72	67	28 (51.8%)	259 (48%)	44 (7.5%)	10 (19%)
Bikdeli, 2021^38^	Medical ward	PE	ROS, ADB	445	15 260	15 705	2.80%	75.2	65.7	235 (52.8%)	7828 (51.3%)	N/A	N/A
Ruiz, 2021^39^	Medical ward	Pneumonia	PS	109	983	1092	10%	70.1	60.7	34 (31.2%)	400 (40.7%)	N/A	N/A
Pieralli, 2021^40^	Medical ward	Pneumonia	PS	111	1009	1120	11%	N/A	N/A	N/A	NA	N/A	N/A
Pucci, 2021^41^	Medical ward	Medical patients	PS	6	134	140	4.30%	86	75	15 (52%)	81 (61%)	N/A	N/A
Jacobs, 2020^42^	Mixed ICU	Medical patients	ROS	131	1358	1489	8.80%	N/A	N/A	N/A	NA	N/A	N/A
Bedford, 2020^43^	Mixed ICU	Medical patients	ROS	541	3126	3667	14.80%	N/A	N/A	N/A	NA	N/A	N/A
Arunachalam, 2020^44^	ICU	Sepsis	ROS	32	1113	1145	2.8	N/A	N/A	N/A	N/A	N/A	N/A
Tang, 2020^45^	Medical ward	PE	ROS	112	3871	3983	2.80%	N/A	N/A	N/A	NA	N/A	N/A
Rombauts, 2020^46^	Medical ward	Pneumonia	ROS	124	1615	1739	5.60%	N/A	N/A	N/A	NA	N/A	N/A
Para, 2020^47^	Medical ward	Medical patients	ROS	196	13 983	14 179	1.40%	N/A	N/A	N/A	N/A	N/A	N/A
Gundlund, 2020^48^	Medical ward	Patients with infections	ROS, ADB	30 307	1 349 629	1 379 936	2.20%	N/A	N/A	N/A	N/A	N/A	N/A
Launey, 2019^49^	ICU	Sepsis	PS	57	204	293	30.4	N/A	N/A	N/A	N/A	N/A	N/A
Pieralli, 2019^50^	Medical ward	Pneumonia	PS	48	420	468	10.30%	82.2	74.7	22 (46%)	221 (52%)	9 (18.8%)	48 (11.4%)
Yoshida, 2018^51^	Mixed ICU	Medical patients	ROS	13	39	52	25%	N/A	N/A	N/A	NA	N/A	N/A
Duarte, 2017^52^	Mixed ICU	Medical patients	PS	24	172	196	12.20%	N/A	N/A	N/A	NA	N/A	N/A
Moss, 2017^53^	Mixed ICU	Medical patients	PS	251	3190	3441	7.3	N/A	N/A	N/A	NA	N/A	N/A
Cheng, 2017^54^	Medical ward	Sepsis	ROS, ADB	1286	65 922	67 208	1.9	78.7	67.3	N/A	N/A	N/A	N/A
Klouwenberg, 2017^11^	ICU	Sepsis	PS	317	1047	1364	23.2	N/A	N/A	108 (44.8%)	914 (45.9%)	N/A	N/A
Krajewska, 2017^55^	Medical ward	PE	ROS	15	303	318	4.70%	N/A	N/A	N/A	NA	N/A	N/A
Violi, 2017^56^	Medical ward	Pneumonia	ROS	46	797	843	5.50%	N/A	N/A	N/A	NA	N/A	N/A
Massera, 2017^57^	Medical ward	Medical patients	ROS	1005	56 056	57 061	1.80%	N/A	N/A	N/A	NA	N/A	N/A
Carrera, 2016^58^	Medical ICU	Medical patients	ROS, ADB	582	7886	8468	6.90%	72	58	250 (43%	3867 (49%)	116 (20%)	63 (8%)
Liu, 2016^59^	ICU	Sepsis	ROS	240	263	503	37.9	77.3	69.5	N/A	NA	89 (37%)	46 (17.5%)
Lewis, 2016^60^	ICU	Sepsis	ROS	26	105	131	19.8	N/A	N/A	N/A	NA	N/A	N/A
Chen, 2015^61^	Medical ICU	Medical patients	ROS, ADB	53	688	741	7.20%	67	56	27	381	24 (45%)	109 (16%)
Guenancia, 2015^62^	ICU	Sepsis	PS	29	37	66	44	71	60.5	N/A	NA	9 (31%)	9 (24.3%)
Koyfman, 2015^63^	ICU	Sepsis	ROS	37	119	156	23.7	N/A	N/A	N/A	NA	N/A	N/A
Ambrus, 2015^64^	ICU	ARDS	PS	21	230	251	8.30%	52	52	13 (46%)	104 (45%)	N/A	N/A
Makrygiannis, 2014^65^	Mixed ICU	Medical patients	PS	15	56	71	21.10%	N/A	N/A	N/A	NA	N/A	N/A
Bajaj, 2014^66^	Medical ward	PE	ROS	15	315	330	4.50%	N/A	N/A	N/A	NA	N/A	N/A
Terzano, 2014^67^	Medical ward	COPD	PS	42	151	193	21.70%	N/A	N/A	N/A	N/A	N/A	N/A
Walkey, 2013^68^	Medical ward	Sepsis	ROS, ADB	4320	44 844	49 164	8.8	N/A	N/A	N/A	NA	N/A	N/A
Soto, 2013^69^	Medical ward	Pneumonia	ROS, ADB	2625	30 064	32 689	8%	N/A	N/A	N/A	NA	N/A	N/A
Walkey, 2011^70^	Medical ward	Sepsis	ROS, ADB	2896	36 200	39 096	7.4	74	66	250 (43%	3867 (49%)	1629 (56.3%)	13 652 (37.7%)
Wells, 2011^71^	ICU	Sepsis	ROS, ADB	328	1138	1466	22.4	N/A	N/A	27	381	N/A	N/A
Mandal, 2011^72^	Medical ward	Pneumonia	ROS, ADB	410	4408	4818	8.50%	N/A	N/A	N/A	NA	N/A	N/A
Ryu, 2010^73^	Medical ward	PE	ROS	5	120	125	4%	N/A	N/A	N/A	NA	N/A	N/A
Morelli, 2009^74^	ICU	Sepsis	PS	5	40	45	11.1	N/A	N/A	N/A	NA	N/A	N/A
Christian, 2008^75^	ICU	Sepsis	ROS	9	175	184	4.9	N/A	N/A	N/A	NA	N/A	N/A
Kindem, 2008^76^	Medical ward	Bacteraemia	ROS	104	568	672	15.50%	N/A	N/A	N/A	N/A	N/A	N/A
Arora, 2007^77^	Mixed ICU	Medical patients	PS	12	19	31	38.70%	N/A	N/A	N/A	NA	N/A	N/A
Musher, 2007^78^	Medical ward	Pneumonia	ROS	5	134	139	3.60%	N/A	N/A	N/A	NA	N/A	N/A
Cuculi, 2006^79^	Medical ward	Alcohol withdrawal	ROS	1	48	49	2%	N/A	N/A	N/A	N/A	N/A	N/A
Calvo-Romero, 2005^80^	Medical ward	PE	ROS	10	144	154	6.50%	N/A	N/A	N/A	NA	N/A	N/A
Seguin, 2004^81^	Mixed ICU	Medical Patients	PS	7	103	110	6.30%	N/A	N/A	N/A	NA	N/A	N/A

ADB, administrative database; ARDS, acute respiratory distress syndrome; ICU, Intensive care unit; PE, pulmonary embolism; PS, prospective study; ROS, retrospective observation study.

^a^Age is mean/median dependent on what was reported in the article. Measures of dispersion (standard deviation, interquartile, or range) were inconsistently reported and are therefore not presented.

### Characteristics of the included studies

All identified papers were published between 2004 and 2026, comprising a total of 3 608 663 patients. The smallest study^77^ included a total of 31 patients, whilst the largest study^48^ included of 1 950 771 patients. 16 of the studies were prospective in nature^11,35,39–41,49,50,52,53,62,64,65,67,74,77,81^ and the remaining 46 retrospective,^6,22–34,37,38,42–48,51,54–61,63,66,68–73,75,76,78–80,82,83^ whereby 17 of these utilized administrative databases.^6,24,25,28,29,31,34,36,38,48,54,58,61,68–72^ The lowest reported cumulative incidence of NOAF was 1.5%^28^ whilst the highest reported cumulative incidence was 44%.^62^ The youngest mean age for both NOAF and controls was 52 years,^64^ the oldest NOAF cohort had a mean age of 86 years^41^ and the oldest control cohort of 75 years.^41^ In every study NOAF patients were older than controls, except for Ambrus *et al.*^64^ where both cohorts were matched for age.

### Cumulative incidence of atrial fibrillation

**Figure 2:**
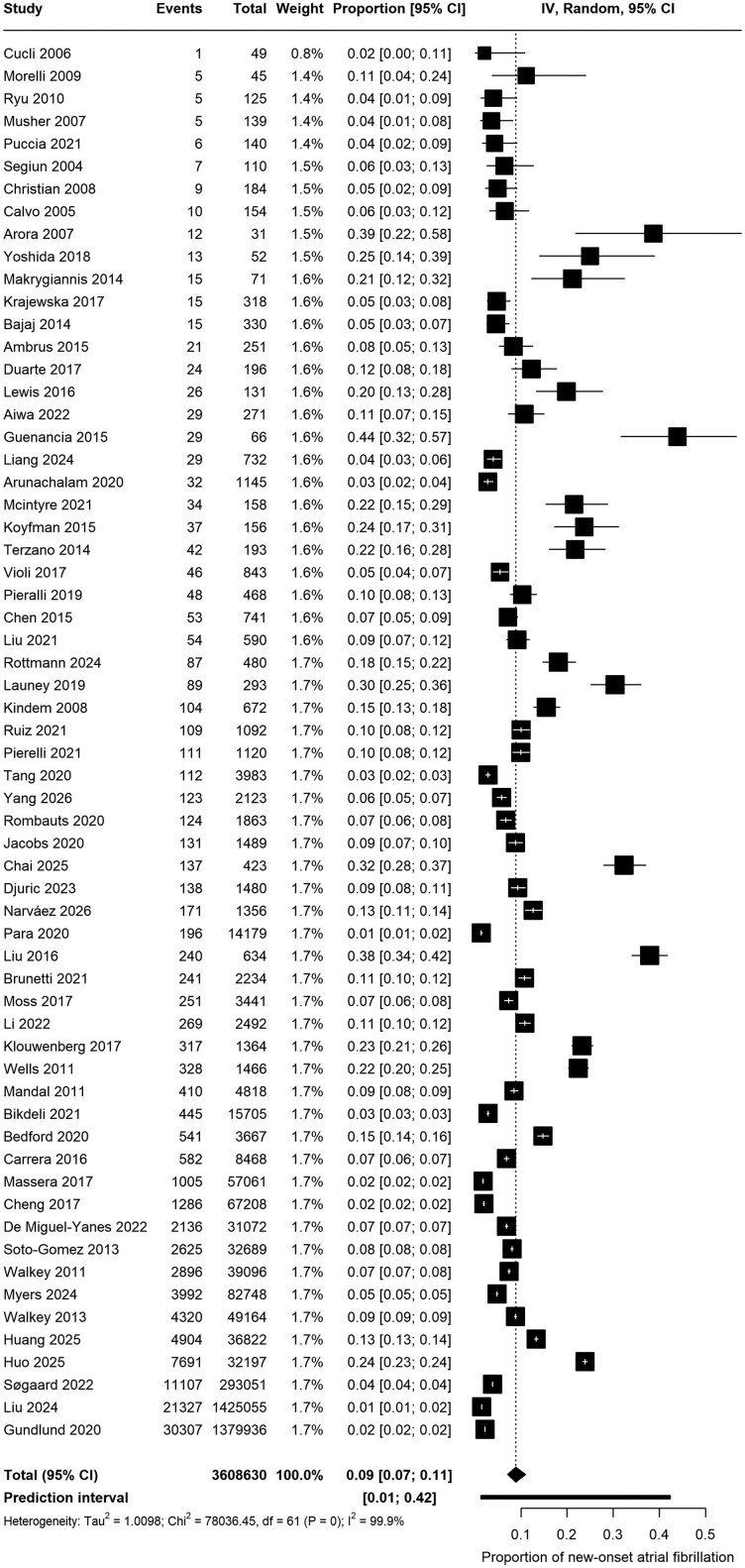
Forest plot showing the pooled incidence of new-onset atrial fibrillation among hospitalized medical patients. Individual study proportions and 95% confidence intervals (CI) are shown for 62 studies (*N* = 3 608 633). The pooled cumulative incidence was 0.09 (95% CI: 0.07–0.11), calculated using a random-effects model (Inverse Variance method with Hartung-Knapp adjustment). Significant heterogeneity was observed (I^2^ = 99.9%, *P* < 0.00001). The prediction interval [0.01; 0.42] indicates the expected range of incidence for a future clinical setting.

The cumulative incidence of NOAF across all studies was 99 474 out of 3 608 630 patients (*Figure 2*). This results in a crude cumulative incidence of 2.4%. The pooled cumulative incidence estimated using a random-effects model with logit transformation was 9% (95% CI 7–11%). This model gives greater weighting to smaller studies. Results demonstrated significant heterogeneity (I^2^ = 99.9%; *P* < 0.0001); reflecting the vastly different methodologies utilized between studies and explaining the difference between the crude and pooled cumulative incidence. The 95% PI from our data was 1–42% suggesting that in future studies there is a 95% chance that the cumulative incidence of NOAF would fall between these values. Leave-one-out sensitivity analysis demonstrated that exclusion of any single study did not materially influence the pooled estimate, with consistent results across all iterations.

A meta-regression was performed to explore potential sources of heterogeneity (*Table 2*).

**Table 2:** Univariate and multivariate meta-regression of moderators of new-onset atrial fibrillation distance

Variable	Univariable OR (95% CI)	*P*-value	*R* ^2^ (%)	Multivariable OR (95% CI)	*P*-value
Year of publication	0.99 (0.95–1.04)	0.72	0.0	1.00 (0.96–1.04)	0.98
ICU setting (vs. hospital)	2.97 (1.99–4.49)	<0.0001	16.0	2.46 (1.22–5.00)	0.013
Prospective study (vs. retrospective)	2.20 (1.30–3.74)	0.004	2.2	1.73 (1.01–2.95)	0.044
Administrative/coded data	0.69 (0.40–1.17)	0.17	1.1	0.90 (0.54–1.52)	0.70
Condition (overall effect)	—	0.0039	∼0.0	—	NS
Log total sample size	0.82 (0.76–0.89)	<0.0001	66.6	—	—

Each row presents the estimated regression coefficient (β), its standard error (SE), 95% confidence interval (CI), and associated *P* value. The exponentiated β is reported as an odds ratio (OR) to help readers interpret out findings. Significance is a *P* < 0.05.

CI, confidence interval; ICU, intensive care unit; OR, odds ratio; SE, standard error; β, regression coefficient.

Our multivariable meta-regression identified ICU setting and prospective study design as independent contributors to between-study heterogeneity, with ICU-based studies [Odds ratio (OR) 2.46, 95% CI 1.22–5.00; *P* = 0.013] and prospective designs (OR 1.73, 95% CI 1.01–2.95; *P* = 0.044) associated with higher effect estimates. Together, these factors contributed to explaining between-study heterogeneity.

In univariable analyses, increasing sample size was strongly associated with lower effect estimates (OR 0.82 per log increase, 95% CI: 0.76–0.89; *P* < 0.0001), explaining a substantial proportion of heterogeneity (*R*^2^ = 66.6%), consistent with potential small-study effects. In contrast, year of publication and use of administrative data were not associated with effect size, and although condition appeared significant in univariable analysis, this did not persist after adjustment.

**Figure 3:**
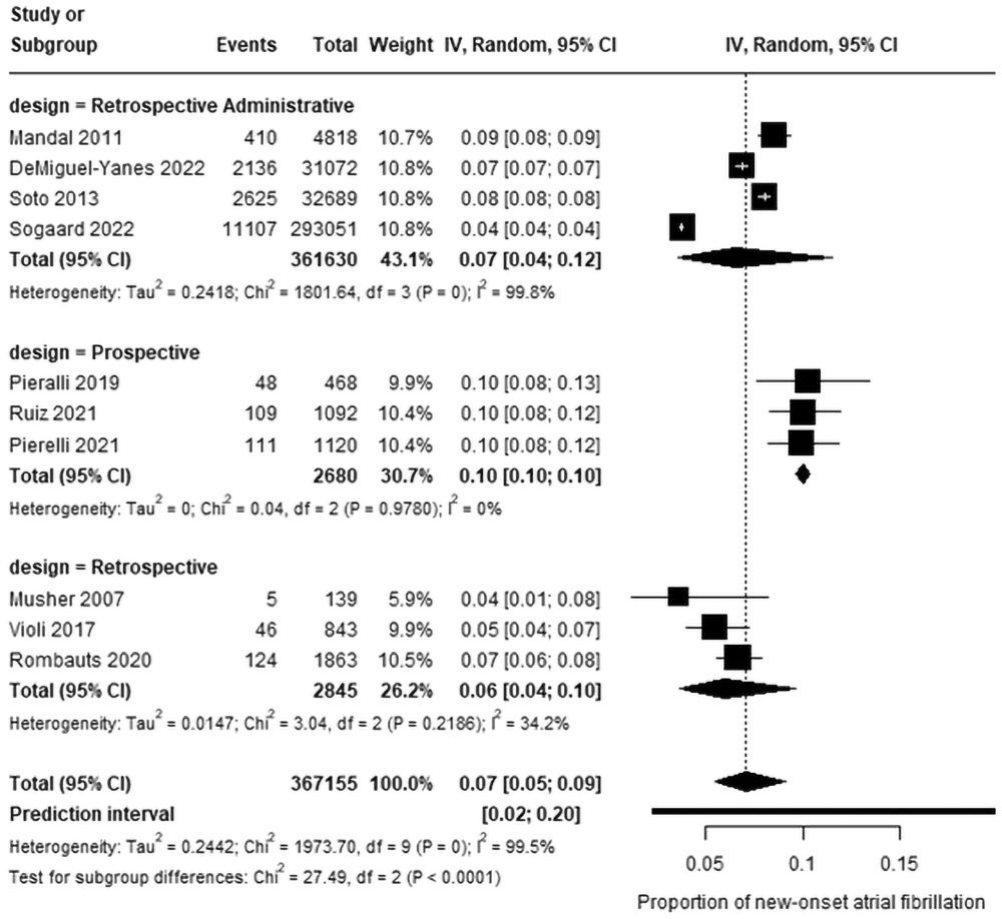
Forest plot of incidence of new-onset atrial fibrillation in patients hospitalized with pneumonia stratified by study design (prospective/retrospective/administrative database). The pooled rate is calculated using a random-effects meta-analysis with the Hartung-Knapp adjustment. 95% CI, 95% confidence interval.

Subgroup analyses stratified by study design demonstrated consistent differences in cumulative incidence. Prospective studies reported higher cumulative incidence of NOAF compared with retrospective studies, whereas administrative database (ADB) studies consistently reported lower estimates. These patterns were observed across multiple cohorts (*Figures 3, 6,* and *7*) and likely reflect differences in monitoring intensity and case ascertainment.

### Subgroup analysis

#### Pneumonia

Across 10 studies,^6,34,39,40,46,50,56,69,72,78^ 16 721 of a total of 367 155 patients developed NOAF during hospitalization with pneumonia (*Figure 3*). The crude cumulative incidence was 4.6% whilst pooled cumulative incidence was 7% (95% CI 5–9%). Significant heterogeneity remained (*I*^2^ = 99.5%), with a wide 95% PI (2–20%). When studies were grouped as prospective and retrospective, heterogeneity was eliminated (*I*^2^ = 0% and 34.2%).

#### Pulmonary embolism

**Figure 4:**
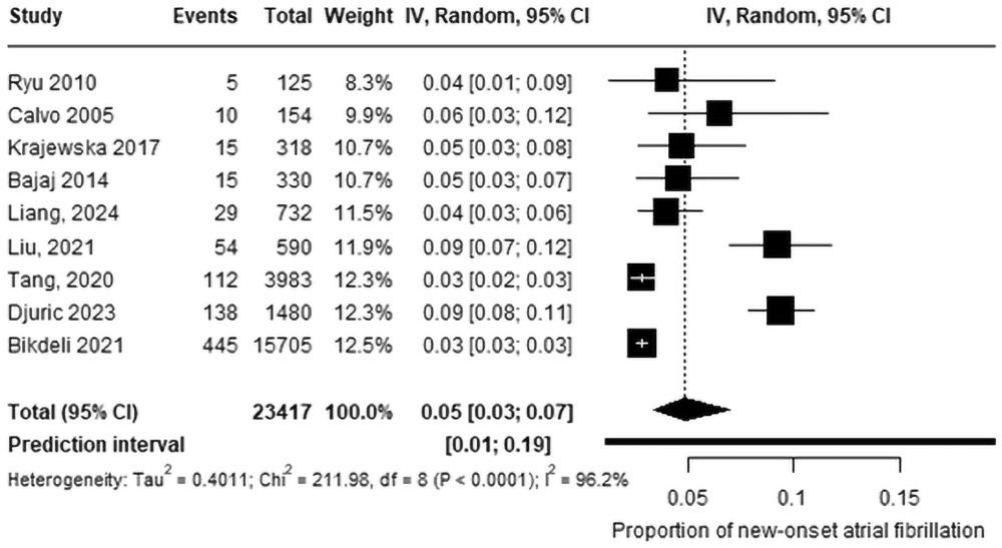
Forest plot demonstrates the prevalence of new-onset atrial fibrillation in patients hospitalized with pulmonary embolism. The pooled rate is calculated using a random-effects meta-analysis with the Hartung-Knapp adjustment. 95% CI, 95% confidence interval.

823 of 23 417 patients (3.5%) with pulmonary embolism developed NOAF across 9 studies^30,31,37,38,45,55,66,73,80^ . The pooled cumulative incidence estimate was 5.01% (95% CI: 3–7%) (*Figure 4*). There remained significant heterogeneity (*I*^2^ = 96.2%) and a wide 95% PI (1–19%), reflecting the expected variability in future studies.

#### Sepsis

**Figure 5:**
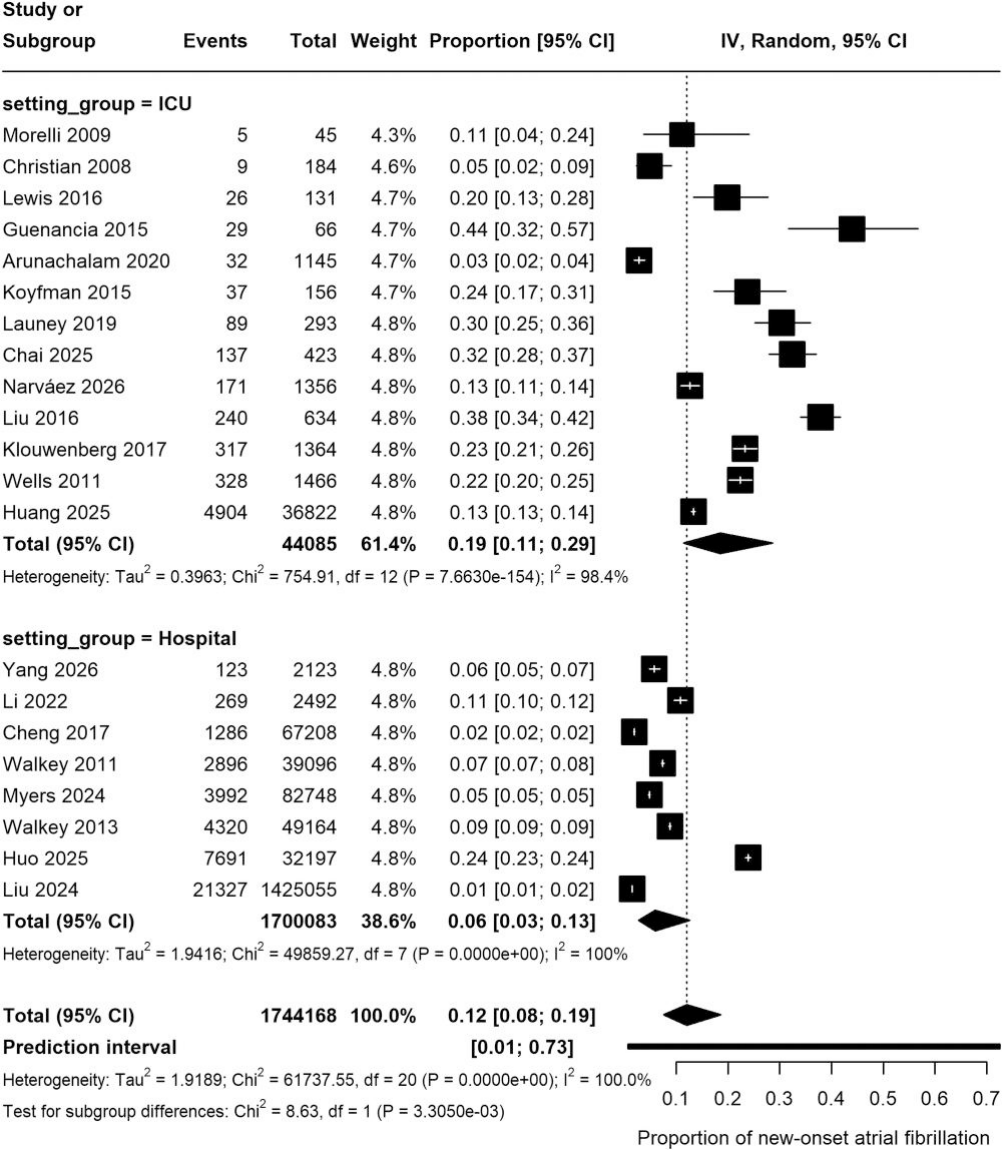
Forest plot showing the pooled incidence of new-onset atrial fibrillation among patients with sepsis. Individual study proportions and 95% confidence intervals (CI) are shown for 21 studies (*N* = 1 744 168), stratified by clinical setting (ICU vs. hospital). The pooled cumulative incidence was calculated using a random-effects model (Inverse Variance method with Hartung–Knapp adjustment). Significant heterogeneity was observed (*I*^2^ = 99.9%, *P* < 0.0001). Prediction intervals are displayed, reflecting the expected range of incidence in future clinical settings. Subgroup analysis compares incidence between ICU and hospital populations.

**Figure 6:**
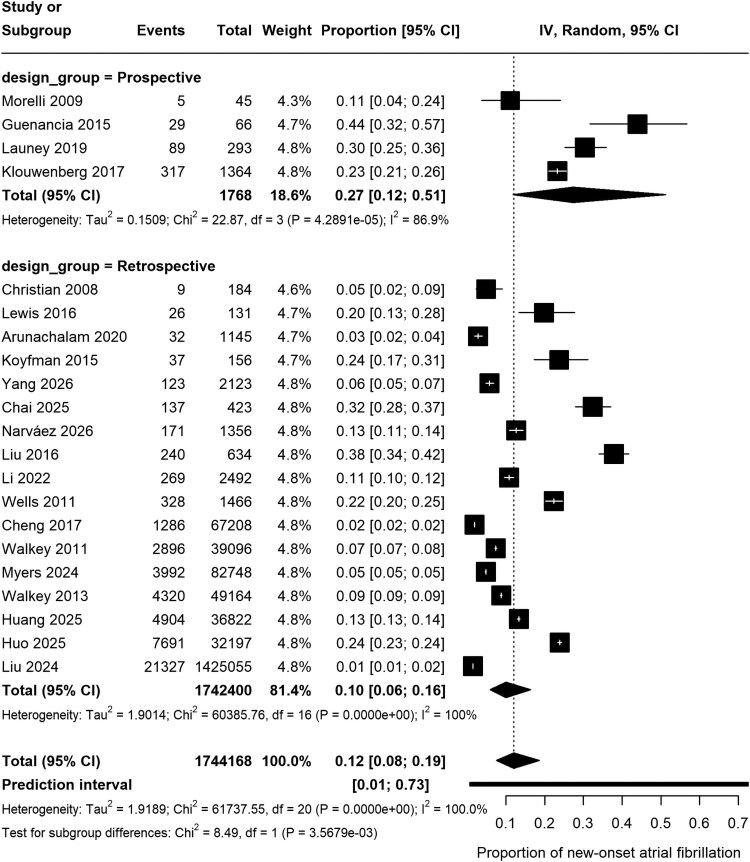
Forest plot showing the pooled incidence of new-onset atrial fibrillation among patients with sepsis. Individual study proportions and 95% confidence intervals (CI) are shown for 21 studies (*N* = 1 744 168), stratified by study design (prospective vs. retrospective). The pooled cumulative incidence was calculated using a random-effects model (Inverse Variance method with Hartung–Knapp adjustment). The incidence was 0.27 (95% CI: 0.12–0.51) in prospective studies and 0.10 (95% CI: 0.06–0.16) in retrospective studies, with an overall pooled estimate of 0.12 (95% CI: 0.08–0.19). Significant heterogeneity was observed in both subgroups (I^2^ = 86.9% for prospective and 100% for retrospective; *P* < 0.0001). The prediction interval for the overall estimate [0.01–0.73] reflects the expected range of incidence in future clinical settings. There was a significant difference between subgroups (*P* = 0.004).

We included a total of 21 studies in this meta-analysis of patients admitted to hospital with sepsis, comprising 1 744 168 patients. We analysed 4 prospective^11,49,62,74^ and 17 retrospective^22–26,28,29,33,44,54,59,60,63,68,70,71,75^ studies. The 8 largest studies were all administrative databases by nature,^24,25,28,29,54,68,70,71^ and these contributed 1 733 756 patients, constituting 99.4% of the total cohort. Eight studies focused on patients admitted to general medical wards^22,25,28,29,33,54,68,70^; in this setting the pooled cumulative incidence of NOAF was 6% (95% CI: 3–11%, *I*^2^ = 100%). Comparatively, 15 studies assessed patients in ICU,^23,24,26,44,49,59,60,62,63,71,74,75,84^ and these demonstrated a significantly higher cumulative incidence of NOAF at 19% (95% CI:11–29%, *I*^2^ = 99.9%). The pooled cumulative incidence of NOAF in both setting was 12% (95% CI: 8–19%). However, there was substantial heterogeneity (*I*^2^ = 100%) and a wide 95% PI (1–73%). There were significantly different cumulative incidences of NOAF between the two groups (*P* < 0.0001), with ICU patients demonstrating a significantly higher NOAF cumulative incidence (*Figure 5*). This is consistent with prior data demonstrating that the severity of sepsis is linked to a higher cumulative incidence of NOAF.^84^ An analysis based on study design (*Figure 6*) demonstrated that prospective studies had higher rates of NOAF [27% (95%: 12–51%)] as compared to retrospective studies [10% (95%: 6–16%)].

#### Cumulative incidence of NOAF in the ICU

**Figure 7:**
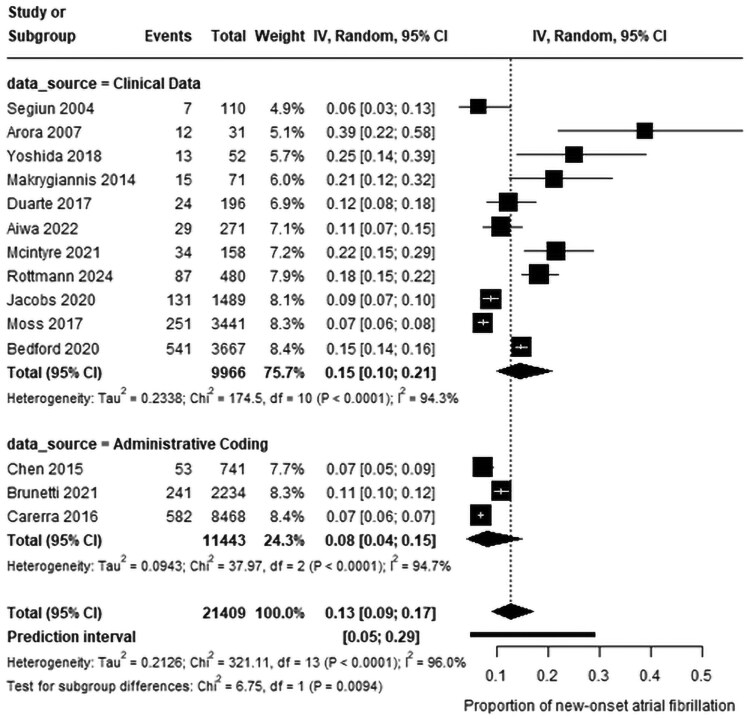
Forest plot showing the pooled incidence of new-onset atrial fibrillation among intensive care unit patients, stratified by study design (administrative vs clinical). The pooled rate is calculated using a random-effects meta-analysis with the Hartung-Knapp adjustment. CI, confidence interval.

2713 patients (12.6%) of patients suffered with NOAF in the ICU setting out of 21 409 patients across 14 studies.^27,32,35,36,42,43,51–53,58,61,65,77,81^ 5 studies were composed of entirely medical patients,^27,32,36,58,61^ whilst the rest of the studies contained a mixture of medical and surgical patients. The pooled cumulative incidence of NOAF was 13% (95% CI: 9–17%) (*Figure 7*) with a wide 95% PI (5–29%). We found that studies employing an ADB reported a cumulative incidence of 8% (95% CI: 4–15%) as compared to studies utilizing clinical data 15% (95%CI: 10–21%). This difference was statistically significant (χ^2^ = 6.75, df = 1, *P* = 0.009).

## Discussion

In this PRISMA-compliant systematic review and meta-analysis, we determined the cumulative incidence of NOAF in 3 608 663 patients admitted to hospital with a variety of medical conditions, whereby a total of 99 474 patients experienced NOAF. This corresponds to a crude cumulative incidence of 2.4%, whilst the pooled cumulative incidence from all studies was 9%.

This review, to the authors’ knowledge, is the largest to define the cumulative incidence of NOAF in various patient cohorts and provides a broad overview of NOAF across various medical conditions and identifies sources of variability between studies.

The included studies featured significant variability in study design, method of NOAF detection and severity of patient illness contributing to wide differences in the reported incidence, ranging from 1.5%^28^ to 44%.^62^ Indeed, the very significant heterogeneity precludes the meta-analysis from providing a single, generalizable cumulative incidence from such diverse data. Our risk of bias assessment revealed that most studies had a low/moderate risk of bias (see Supplementary material online, *Table S2*).

In patients with pneumonia, the relatively high incidence of NOAF is likely multifactorial, reflecting systemic inflammation, autonomic imbalance, and hypoxaemia, all of which may promote atrial electrical instability. Acute infection may also exacerbate underlying atrial vulnerability through haemodynamic stress and volume shifts, particularly in older patients with cardiovascular comorbidities.^85^ The marked heterogeneity observed across studies likely reflects differences in disease severity, patient populations, AF detection methods, and monitoring intensity. In patients with sepsis, the incidence of NOAF is likely driven by a combination of systemic inflammatory activation and acute physiological stress. Elevated circulating cytokines may contribute to atrial electrical and structural remodelling, promoting arrhythmogenesis. This is compounded by autonomic imbalance, with heightened sympathetic activation, as well as haemodynamic instability, electrolyte disturbances, and myocardial dysfunction, all of which may increase susceptibility to NOAF. The marked heterogeneity observed across studies likely reflects differences in illness severity, patient populations, and methods of AF detection, particularly given the heavy contribution of large administrative datasets with lower sensitivity for arrhythmia detection.^86^ Our findings are consistent with prior literature. For example, in patients with pneumonia, the pooled cumulative incidence of NOAF in our analysis (7%) is comparable to the 7.6% reported by Corica *et al.*^87^ This study reinforces and extends a prior attempt to answer this question by Mcintyre *et al.*^17^ in 2019, who identified 36 studies but did not pool the cumulative incidence estimate due to the substantial heterogeneity. Whilst we acknowledge the considerable heterogeneity in our own study, we opted to synthesize the data using a random-effects model, which is designed to account for such variability. Furthermore, we used the Hartung-Knapp adjustment to incorporate uncertainty around between-study variance. However, it should be stated that within the limitations of these models, the pooled cumulative incidence should not be interpreted as a universal figure, but rather as a summary reflecting the broad range reported across the multiple studies.

We acknowledge, as Mcintyre *et al.*^17^ did, that a single pooled estimate is not generalizable given the diversity of this data, nevertheless, our pooled result serves as a rough benchmark. Furthermore, this analysis offers a more integrated understanding of the burden of NOAF, especially within specific disease cohorts, while recognizing the limitations imposed by study-level differences. Our analysis offers a deeper understanding of where the heterogeneity originates from via our multivariate meta-regression which identified ICU setting and prospective design as independent contributors to between-study variability, with both associated with higher reported effect estimates. While these factors accounted for a meaningful proportion of heterogeneity, a substantial degree of variability remained unexplained. Notably, increasing sample size was strongly associated with lower effect estimates in univariable analysis, suggesting the potential influence of small-study effects. We believe this novel aspect, and the fact we identified 62 articles and analysed specific disease cohorts, extends the previously published work.

Mcintyre *et al.*^17^ identified monitoring frequency as highly predictive of NOAF diagnosis, in their post-hoc analyses continuous cardiac monitoring was associated with a higher cumulative incidence of NOAF. In our analysis, ICU-based studies, which inherently contain the highest proportion of continuous cardiac monitoring, displayed the highest cumulative incidence of NOAF. The high incidence of NOAF observed in critically ill patients is likely multifactorial: Driven by systemic inflammatory activation, heightened sympathetic tone, and haemodynamic instability promoting atrial electrical instability. In addition, electrolyte disturbances, atrial stretch due to fluid shifts and increased filling pressures, and myocardial dysfunction may further predispose to atrial arrhythmogenesis in this population.^10^ This may also be influenced by the higher disease severity linked to ICU admission. In our study the highest individual level NOAF cumulative incidence of 44% was reported by Guencia *et al.*^62^ They used 7-day Holter monitoring in patients admitted with septic shock and demonstrated that aggressive monitoring leads to higher reported incidence of NOAF. In contrast, Liu *et al.*^28^ performed a search using an ADB and reported a 1.5% cumulative incidence of NOAF. These studies highlight the importance of using continuous ECG monitoring as the gold standard detection method. Studies reporting a lower cumulative incidence of NOAF are likely reporting the minimum cumulative incidence caught during routine care, whilst studies reporting higher cumulative incidence likely reflect the maximum cumulative incidence by detecting asymptomatic and undiagnosed episodes of NOAF.

The prediction interval converts the high heterogeneity into a clinically useful range. The 95% prediction interval is between 1 and 42%, suggesting that any future study is likely to report results within this range. In clinical care settings this may translate to 20% of critically ill patients admitted to < ICU with sepsis being diagnosed with NOAF, whilst in a general medical ward undifferentiated medical patients only a 3% cumulative incidence may be reported. Our results highlight that the variability between studies is much greater than the variability within studies. Overall, our data suggest that the cumulative incidence of NOAF is context specific and dependent on the clinical scenario assessed.

Historically, NOAF during acute medical illness was labelled as ‘secondary AF’ and would prompt a search for a treatable precipitant.^7^ However, these patients remained at high risk of long-term complications.^8^ Therefore, we did not look at cumulative incidence of AF reoccurrence post discharge as this has become largely clinically irrelevant. There is increasing recognition that these episodes of NOAF are the manifestation of a tendency towards AF at least in a significant proportion of patients. Empirical treatment of AF should therefore be offered. This is backed by a 2023 American Heart Association scientific statement emphasizing that cumulative incidence is highly variable, in keeping with our findings and that these patients should generally be offered anticoagulation depending on traditional risk scoring schemata due to the high burden of recurrence.^9^ Whilst this is applicable to the general hospitalized medical patient, it may not be generalizable to patients in ICU, where adrenergic overstimulation and cytokine storm may be the predominant trigger and result in a single isolated episode.^10^

Management of NOAF in hospitalized patients is typically focused on rate or rhythm control alongside treatment of the underlying precipitating condition. While catheter ablation is an established therapy in selected patients with AF, its role in the acute hospital setting remains limited, although increasing, and is generally reserved for selected patients following clinical stabilization, reflecting both the acute clinical context and emerging evidence suggesting higher procedural risk when performed early.^88^

Moving forward, there is a need for a high quality, prospective study employing continuous cardiac monitoring. The ‘Intelligent Monitoring to Predict Atrial Fibrillation’ (NCT06600620) is prospective observational study being performed in two tertiary hospitals in Liverpool, United Kingdom. This study aims to recruit 1200 patients, deemed high risk for the development of NOAF over 4 years and monitor them using a cardiac device capable of recording ECGs at 100 Hz a second for up to 7 days during hospitalization. This will reflect the best attempt at quantifying the cumulative incidence of NOAF in hospitalized patients.

### Strength and limitations

We searched multiple bibliographic databases using a systematic strategy to identify all relevant studies. Two authors independently screened studies for inclusion and exclusion criteria and extracted the data. We performed subgroup and sensitivity analyses.

Our study should be viewed within the context of its limitations. First, the most significant limitation to our findings is the very high heterogeneity (*I*^2^ = 99.9%) which challenges the usefulness of the pooled cumulative incidence. We attempted to identify the source of heterogeneity using a multi-variate meta-regression and performed a subgroup analysis stratified by clinical condition, study design, and care setting to provide more in-depth results. We have demonstrated that the cumulative incidence of NOAF is context and disease specific, with higher estimated observed in prospective study and critically ill populations, likely reflecting differences in monitoring intensity, disease severity, and case ascertainment. Despite this, we are unable to provide a single summary result that can be extrapolated to the general medical patient. As such, the pooled cumulative incidence should be interpreted as a summary across diverse clinical scenarios rather than a single generalizable estimate applicable to all hospitalized patients. The wide prediction intervals further highlight this variability and provide a more clinically meaningful range of expected values in different settings. This is because the general medical patient population is by itself a very heterogenous population, encompassing a wide range of clinical conditions with differing risks of NOAF. Due to the substantial clinical and methodological heterogeneity across studies, formal assessment of small-study effects (e.g. funnel plot asymmetry or Egger’s test) was not performed, as such methods may yield misleading results under these conditions. Variation in hospitalization duration across studies may have influenced the observed cumulative incidence of NOAF, as longer admissions increase the likelihood of arrhythmia detection. Furthermore, differences in AF diagnostic criteria and case definitions across studies, including reliance on ECG confirmation, continuous monitoring, or administrative coding, may have contributed to variability in reported incidence. In studies utilizing administrative databases, misclassification bias may have occurred due to coding inaccuracies or reduced sensitivity for detecting transient or asymptomatic AF. Finally, the use of study-level variables in meta-regression introduces the possibility of ecological bias, whereby associations observed at the study level may not reflect individual patient-level relationships. Second, our search was conducted in the English language and hence it is likely that we have missed some relevant articles, however given the large number of analysed studies this would be unlikely to change our results. Third, there is no search term for ‘medical hospitalization’ and hence our search at best acts as a proxy for conditions that may result in hospitalization for a medical condition. Additionally, the NOS is not validated for cumulative incidence detection and hence the reliability of this tool for our included studies is questionable. Furthermore, included studies employed various methods of diagnosing NOAF, and this was recognized on a previous study to influence NOAF cumulative incidence.^17^ Additionally, our inclusion of administrative databases and national registries acts as a limitation. In such datasets, there is a possibility of overlapping patient populations across studies, particularly when similar data sources or healthcare systems are used over comparable time periods. Due to the use of aggregated published data, it was not possible to identify or exclude duplicate patient inclusion at an individual level. While we attempted to minimize this risk by reviewing study characteristics including geographic location, study period, and data source, residual overlap cannot be excluded and may have contributed to the observed heterogeneity. Furthermore, we also limited our search to studies published in English and from the year 2000 onwards, which may have introduced selection bias and limited the inclusion of potentially relevant earlier or non-English studies. However, given the large number of included studies and the consistency of findings across diverse populations and settings, it is unlikely that inclusion of such studies would have materially altered the overall conclusions. Finally, our study focuses on the cumulative incidence of NOAF and does not evaluate its prognostic implications, including mortality, thromboembolic risk, or recurrence. While clinically important, these outcomes were beyond the scope of the present analysis. Furthermore, the observational nature of the included studies and the lack of consistent adjustment for baseline differences limit the ability to draw causal inferences regarding the prognostic impact of NOAF. Future prospective studies are needed to address these questions.

## Conclusion

The cumulative incidence of NOAF during acute medical illness varies and is dependent on (I) severity of illness and (II) methodology of identifying it. Efforts to pool studies are limited by variations in study design, patient characteristics and illness severity. There is a need for high quality prospective data derived from hospitalized patients to identify the cumulative incidence of NOAF.

## Supplementary Material

oeag080_Supplementary_Data

## Data Availability

All data supporting the findings of this study are included within the manuscript and its supplementary materials.

## Appendix 1. Systematic review search strategy

**Table:**

MEDLINE and EMBASE via Ovid
1. Atrial Fibrillation/
2. [Atrial* adj2 (Fibrillat* or flutter*)].tw.
3. AF.tw.
4. (tachycardia or tachyarrhythmia or arrhythmia or supraventricular).tw.
5. 1 or 2 or 3 or 4
6. afib.ti,ab,kw,kf.
7. a fib.ti,ab,kw,kf.
8. [(new* or recent*) adj1 diagno*].tw.
9. (onset* or new*).tw.
10. 8 or 9
11. (acute adj3 stress*).ti,ab,kw,kf.
12. infection.sh.
13. infection*.ti,ab,kw,kf.
14. exp sepsis/
15. sepsis.ti,ab,kw,kf.
16. septic*.ti,ab,kw,kf.
17. blood adj3 poison*).ti,ab,kw,kf.
18. bacteremia*.ti,ab,kw,kf.
19. soft tissue infections.sh.
20. abscess.sh.
21. respiratory tract infections.sh.
22. Heart failure.ti,ab,kw,kf.
23. exp endocarditis, bacterial/
24. endocarditi* adj3 bacterial).ti,ab,kw,kf.
25. urinary tract infections.sh.
26. Gastroenteritis.ti,ab,kw,kf.
27. cellulitis.ti,ab,kw,kf.
28. osteomyelitis.ti,ab,kw,kf.
29. diverticulitis.ti,ab,kw,kf.
30. exp pneumonia/
31. pneumoni*.ti,ab,kw,kf.
32. bronchopneumonia*.ti,ab,kw,kf.
33. COPD.ti,ab,kw,kf.
34. lung disease*.ti,ab,kw,kf.
35. asthma*.ti,ab,kw,kf.
36. (pulmonary adj3 embolism*).ti,ab,kw,kf.
37. venous thromboembolism.sh.
38. respiratory distress syndrome, adult.sh.
39. exp shock/
40. [circulat* adj3 (fail* or collaps*)].ti,ab,kw,kf.
41. severe acute respiratory syndrome.sh.
42. (respiratory adj3 syndrome*).ti,ab,kw,kf.
43. exp empyema/
44. exp alcohol related disorders/
45. Exp.stroke
46. Exp,Transient ischaemic attack
47. accidental falls.sh.
48. fall*.ti,kw,kf.

Search strategy employed in Medical Literature Analysis and Retrieval System Online (MEDLINE) and Excerpta Medica Database (EMBASE). Both databases were searched for articles published between 01 January 2000 and 10 March 2026 (search date).
