# Supplementary material for: The cumulative incidence of atrial fibrillation in the hospitalized medical patient: a systematic review and meta-analysis
Source: Eur Heart J Open. 2026 May 22;6(3):oeag080. doi: 10.1093/ehjopen/oeag080 (PMC13252544; doi:10.1093/ehjopen/oeag080)
Supplement: oeag080_Supplementary_Data [file oeag080_supplementary_data.docx]

Supplementary table 1

| **Selection (Max 4 points)**  Sample representation of average patient for study condition  1 point – Sample representative of average patient for studied condition  1 point – AF at baseline appropriately excluded using chart review, ECG, or validated method  1 points - Objective diagnosis using ECG, telemetry, or ICD codes validated in hospital records  1 point - All patients followed through entire hospital stay or until AF occurred |
| --- |
| **Comparability (Max 2 points)**  2 points – Study attempts to adjust for key factors  1 point – Study does not attempt to adjust for key factors |
| **Outcome (Max 3 points)**  1 point - Incident AF confirmed by ECG, rhythm strip, or cardiologist diagnosis  1 point - Incidence clearly reported with numerator/denominator and time frame; appropriate methods used  1 point – The study reported descriptive statistics of both the atrial fibrillation and control population |

Legend – ECG – electrocardiogram, ICD – International Classification of Diseases

Supplementary table 2 – Bias assessment

| Author, year | Selection | Comparability | Outcome | Total NOS | Risk of bias |
| --- | --- | --- | --- | --- | --- |
| Yang, 2026 | 1 | 2 | 3 | 6 | Moderate risk |
| Narváez, 2026 | 3 | 2 | 3 | 8 | Low risk |
| Huang, 2025 | 2 | 1 | 2 | 5 | Moderate risk |
| Huo, 2025 | 2 | 2 | 2 | 5 | Moderate risk |
| Chai, 2025 | 3 | 2 | 3 | 8 | Low risk |
| Liu, 2024 | 4 | 2 | 3 | 9 | Low risk |
| Myers, 2024 | 4 | 2 | 3 | 9 | Low risk |
| Li, 2022 | 4 | 2 | 3 | 9 | Low risk |
| Arunachalam, 2020 | 4 | 2 | 3 | 9 | Low risk |
| Launey, 2019 | 4 | 2 | 3 | 9 | Low risk |
| Cheng, 2017 | 3 | 2 | 3 | 8 | Low risk |
| Klouwenberg, 2017 | 4 | 2 | 3 | 9 | Low risk |
| Liu, 2016 | 4 | 2 | 3 | 9 | Low risk |
| Lewis, 2016 | 3 | 1 | 3 | 7 | Low risk |
| Guenancia**,** 2015 | 4 | 2 | 3 | 9 | Low risk |
| Koyfman, 2015 | 3 | 1 | 3 | 7 | Low risk |
| Walkey, 2013 | 4 | 2 | 3 | 9 | Low risk |
| Walkey, 2011 | 4 | 2 | 3 | 9 | Low risk |
| Wells, 2011 | 3 | 1 | 3 | 7 | Low risk |
| Morelli, 2009 | 3 | 1 | 3 | 7 | Low risk |
| Christian, 2008 | 4 | 0 | 3 | 7 | Low risk |
| Pneumonia | | | | | |
| DeMiguel-Yanes 2022 | 2 | 2 | 2 | 6 | Moderate risk |
| Søgaard 2022 | 3 | 1 | 2 | 8 | Low risk |
| Ruiz 2021 | 3 | 2 | 3 | 8 | Low risk |
| Pierelli 2021 | 3 | 2 | 3 | 8 | Low risk |
| Rombauts 2020 | 2 | 1 | 3 | 6 | Moderate risk |
| Pieralli 2019 | 3 | 2 | 3 | 8 | Low risk |
| Violi 2017 | 1 | 1 | 1 | 3 | High risk |
| Soto 2013 | 2 | 1 | 2 | 5 | Moderate risk |
| Mandal 2011 | 2 | 1 | 2 | 5 | Moderate risk |
| Musher 2007 | 1 | 0 | 1 | 2 | High risk |
| Pulmonary embolism | | | | | |
| Liang 2024 | 2 | 2 | 3 | 7 | Low risk |
| Djuric 2023 | 4 | 2 | 3 | 9 | Low risk |
| Liu 2021 | 3 | 2 | 3 | 8 | Low risk |
| Bikdeli 2021 | 2 | 2 | 3 | 7 | Low risk |
| Tang 2020 | 3 | 2 | 2 | 7 | Low risk |
| Krajewska 2017 | 3 | 2 | 2 | 7 | Low risk |
| Bajaj 2014 | 3 | 2 | 2 | 8 | Low risk |
| Ryu 2010 | 4 | 2 | 2 | 8 | Low risk |
| Calvo 2005 | 3 | 2 | 3 | 8 | Low risk |
| Intensive care | | | | | |
| Rottmann, 2024 | 3 | 2 | 2 | 7 | Low risk |
| Aiwa, 2022 | 3 | 2 | 1 | 6 | Moderate risk |
| Mcintyre, 2021 | 2 | 2 | 3 | 7 | Low risk |
| Brunetti, 2021 | 2 | 2 | 3 | 7 | Low risk |
| Jacobs, 2020 | 2 | 2 | 2 | 6 | Moderate risk |
| Bedford, 2020 | 2 | 2 | 3 | 7 | Low risk |
| Yoshida, 2018 | 2 | 2 | 2 | 6 | Moderate risk |
| Duarte, 2017 | 2 | 2 | 2 | 6 | Moderate risk |
| Moss, 2017 | 2 | 2 | 2 | 6 | Moderate risk |
| Carrera, 2016 | 3 | 2 | 2 | 7 | Low risk |
| Chen, 2015 | 3 | 2 | 3 | 8 | Low risk |
| Makrygiannis, 2014 | 2 | 2 | 3 | 7 | Low risk |
| Arora, 2007 | 2 | 2 | 3 | 7 | Low risk |
| Segiun, 2004 | 2 | 2 | 3 | 7 | Low risk |
| Other | | | | | |
| Pucci, 2021 | 2 | 2 | 3 | 7 | Low risk |
| Para, 2020 | 2 | 2 | 3 | 7 | Low risk |
| Gundlund, 2020 | 2 | 2 | 2 | 7 | Low risk |
| Massera, 2017 | 3 | 2 | 2 | 7 | Low risk |
| Ambrus, 2015 | 4 | 2 | 2 | 9 | Low risk |
| Terzano, 2014 | 3 | 2 | 2 | 7 | Low risk |
| Kindem, 2008 | 2 | 1 | 2 | 5 | Moderate risk |
| Cuculi, 2006 | 1 | 1 | 2 | 4 | Moderate risk |
